# Characterization of the Complete Mitochondrial Genome of *Ostertagia trifurcata* of Small Ruminants and its Phylogenetic Associations for the Trichostrongyloidea Superfamily

**DOI:** 10.3390/genes10020107

**Published:** 2019-01-31

**Authors:** Awais Ali Ahmad, Xin Yang, Ting Zhang, Chunqun Wang, Caixian Zhou, Xingrun Yan, Mubashar Hassan, Muhammad Ikram, Min Hu

**Affiliations:** 1 State Key Laboratory of Agricultural Microbiology, Key Laboratory for the Development of Veterinary Products, Ministry of Agriculture, College of Veterinary Medicine, Huazhong Agricultural University, Wuhan 430070, China; awais@webmail.hzau.edu.cn (A.A.A.); xinyang@webmail.hzau.edu.cn (X.Y.); tingzhang1@webmail.hzau.edu.cn (T.Z.); wangchunqun@webmail.hzau.edu.cn (C.W.); ZCX19920102@webmail.hzau.edu.cn (C.Z.); yanxingrun@webmail.hzau.edu.cn (X.Y.); mubashar.hassan@webmail.hzau.edu.cn (M.H.); 2 Statistical Genomics Lab, College of Plant Science and Technology, Huazhong Agricultural University, Wuhan 430070, China; ikramuaf35@outlook.com

**Keywords:** *Ostertagia trifurcata*, mitochondrial genome, mitochondrial DNA, phylogenetic analysis

## Abstract

The complete mitochondrial (mt) genome of *Ostertagia trifurcata*, a parasitic nematode of small ruminants, has been sequenced and its phylogenetic relationship with selected members from the superfamily Trichostrongyloidea was investigated on the basis of deduced datasets of mt amino acid sequences. The entire mt genome of *Ostertagia trifurcata* is circular and 14,151 bp in length. It consists of a total of 36 genes comprising 12 genes coding for proteins (PCGs), 2 genes for ribosomal RNA (rRNA), 22 transfer RNA (tRNA) genes and 2 non-coding regions, since all genes are transcribed in the same direction. The phylogenetic analysis based on the concatenated datasets of predicted amino acid sequences of the 12 protein coding genes supported monophylies of the Haemonchidae, Dictyocaulidae and Molineidae families, but rejected monophylies of the Trichostrongylidae family. The complete characterization and provision of the mtDNA sequence of *Ostertagia trifurcata* provides novel genetic markers for molecular epidemiological investigations, systematics, diagnostics and population genetics of *Ostertagia trifurcata* and its correspondents.

## 1. Introduction

Gastrointestinal parasites cause major economic losses to the livestock industry all over the world [1]. Among these parasites, *Ostertagia* spp., which is a reddish brown worm present in the abomasum of ruminants, is a major cause of parasitic gastritis (ostertagiosis) worldwide, particularly in temperate climates. *Ostertagia* spp. is considered to be among the most common gastrointestinal nematodes of ruminants [2]. Postmortem examination of small ruminants revealed a high infection rate in goats in China [2]. More than 15 *Ostertagia* species have been reported in small ruminants [3,4,5]. Among them, *Ostertagia trifurcata* (*O. trifurcata*) is distributed widely and has a lifecycle similar to *Haemonchus contortus*, another important parasitic nematode of small ruminants. Animals infected with *Ostertagia* spp. show the presence of eggs in fecal samples 15–17 days after infection [6]. Importantly, *Ostertagia* spp. is prevalent in both temperate and cold climates [7]. Heavy infection leads to emaciation, anemia, intermittent constipation and even death in extreme cases [8]. In China, it is one of the most predominant nematodes of ruminants and contributes to substantial financial losses [9].

Mitochondria are a subcellular organelle with important biochemical functions. This organelle is the powerhouse of the eukaryotic cell. The mitochondrial (mt) genome is located within the organelle, independent of the nuclear genome but with a closer relationship to each other. The mt genome is maternally inherited, and has stable genes, a variable gene arrangement and a faster gene evolution rate [10,11,12]. These features make them widely applicable in epidemiological studies, population genetics and phylogenetic relationships at different taxonomic levels [13,14,15,16,17].

The current hypothesis of Trichostrongylidae’s phylogeny was based on ecological and morphological characteristics along with the sequence analysis of small subunit (SSU) rRNA genes [6,18]. Moreover, reconstructions of phylogenetic relationships among Trichostrongylidae nematodes have been performed using the mt genome sequences [19]. Regardless of the advancements, there is still ambiguity relating to the phylogenetic relationships among Trichostrongylidae nematodes. Some previous studies were indicative of Trichostrongylidae monophyly [20,21], whereas other studies support a contrary argument and are suggestive of a sister relationship among Trichostrongylidae, Haemonchidae and Cooperiidae [18,22,23]. Insufficient perseverance at higher levels of taxonomy with dissimilar datasets of DNA, as well as the utilization of distinct methods for inference may result in such inconsistent results. Even though Trichostrongylidae is a large family of nematodes, the number of complete mt genomes sequenced to date are limited [19]. The enrichment of information on the mt genome of helminths, especially those infecting small ruminants, is required to augment database and species characterization, which provides valuable information for future studies on the identification of species, phylogenetic analysis and genetic diversity. There is very limited availability of genomic data on the mt genome of members of *Ostertagia* genus. This lack of adequate knowledge about the mt genomes of nematodes is a key limitation for studies of the phylogenetic relationship of Trichostrongylidae.

Keeping in view the background and connotation of *O. trifurcata*, the current study intended to determine the mt genome composition of *O. trifurcata* and a reconstruction of the phylogenetic relationship of the Trichostrongyloidea superfamily using these mtDNA sequences.

## 2. Materials and Methods

### 2.1. Collection of Worms and Extraction of DNA

The adult worms from the abomasum of naturally-infected domesticated sheep and goats in Luotian, Hubei, P.R. China were collected. The collected worms were subsequently washed in 0.9% sodium chloride solution and identified as *Ostertagia* based on their morphological characteristics. Samples were then washed with phosphate buffered saline (PBS), fixed in 70% ethyl alcohol, and stored at −20 °C until next use. It was challenging to attain precise morphological characteristics of, so molecular identification was carried out. For the extraction of the total genomic DNA from single worm *Ostertagia* samples, Sodium dodecyl (SDS)-proteinase K treatment was performed, trailed by purification using mini column (Wizard® SV Genomic DNA Purification System, Promega).

### 2.2. Amplification of the ITS-2 of Ostertagia trifurcata

To identify the organism, the ITS-2 region was amplified and then sequenced according to a previously described method [24]. The universal primers NC5 and NC2 (Table 1) were used for the amplification of the ITS-2 region. A total volume of 20 µL was prepared including DNA template, primers and PCR premix (Takara, Dalian, China). The conditions used for PCR amplification were initially 94 °C for 5 min followed by 35 cycles of 94 °C for 30 seconds, 50 °C for 30 seconds and 72 °C for 1 min, final extension at 72 °C for 10 min, and the reaction was stopped at 20 °C for 5 min.

### 2.3. Amplification of Long Fragments and Sequencing

The primers used in amplifying long overlapping fragments of mitochondrial genome were relative to their conserved regions (Table 1) [25]. Long-range PCR was used to amplify the whole mt genome of *O. trifurcata* in four overlapping fragments with locations of amplicons between *rrn*S and *cyt*b (~3 kb), *cyt*b and *cox*1 (~4 kb), *cox*1 and *rrn*L (~3 kb) and *rrn*L and *rrn*S (~5 kb). The long PCRs were performed by making a total volume of 50 µl per amplicon, with the reaction mixture containing 34.75 µL dH_2_O, 5 µL of 10× Thermopol reaction buffer (Biolabs, New England), 10 mM of each dNTP (Takara, Dalian, China), 1.25U LATaq (Takara, Dalian, China), 2 µM of each primers (TsingKe, Beijing, China) and 2 µL of genomic DNA in a thermocycler (Biometra, Göttingen, Germany). The PCR conditions for the amplification were initiated by denaturation at 94 °C for 5 min, followed by 35 cycles of denaturation for 30 seconds at 94 °C, annealing for 30 seconds at 50 °C, extension for 5 min at 60 °C, with 7 min of final extension at 60 °C, and finally the reaction was stopped at 4 °C. The obtained amplicons were then cloned into pGEM-T-Easy vector (Promega, USA), which were sequenced (Sangon BioTech company, Shanghai, China) employing a strategy of primer-walking [26]. The complete mitochondrial genome of *O. trifurcata* (GenBank accession no. MK227249) was thus obtained.

### 2.4. Gene Annotation and Sequence Analysis

The mt genome annotation was performed by implementing a methodology similar to Ascaridomorph nematodes [27]. The assembly of sequences was carried out manually and the assembled sequences were subsequently aligned against the entire mt genome sequences of the reference species (*Teladorsagia circumcincta*, accession number GQ888720) to identify gene boundaries. The Open Reading Frame Finder (<http://www.ncbi.nlm.nih.gov/gorf/gorf.html>) and DOGMA tool (http://dogma.ccbb.utexas.edu/index.html) were used to analyze the open reading frames using the invertebrate mitochondrial code with further comparison performed using other enoplid nematodes. The MEGA5 software was used to select the invertebrate mt genetic code for the translation of individual genes into amino acid sequences. The amino acid sequences of other nematodes were then aligned with the resulting sequences of amino acids inferred for the mt genes using Clustal × 1.83. Based on the pairwise comparison, amino acid identity (%) was also calculated for homologous genes. Codon usage was inspected whereby the genetic codons were split into rich GC codons, rich AT codons and neutral codons based on the relationships among codon families, the occurrence of amino acids and composition of nucleotides. To examine the rRNA genes, presumed secondary structures of tRNA genes were recognized using ARWEN (http://mbio-serv2.mbioekol.lu.se/ARWEN/) [28,29] as well as visual inspection [30].

### 2.5. Phylogenetic Analysis on Basis of the Dataset of Amino Acid Sequences

Individual genes of the *O. trifurcata* mt genome were translated to obtain amino acid sequences that were then integrated to form a single alignment. These sequences were aligned with other deduced sequences of amino acids from already-published mt genomes. Selective nematodes were representatives for comparison with the superfamily Trichostrongyloidea, featuring family Trichostrongylidae (*Trichostrongylus vitrinus*, NC_013807; *Trichostrongylus axei*, NC_013824; *Teladorsagia circumcincta*, NC_013827; *Marshallagia marshalli*, MG011723) [19,31], Molineidae family (*Nematodirus oiratianus*, NC_024639, and *Nematodirus spathiger*, NC_024638) [22], Cooperiidae family (*Cooperia oncophora*, NC_004806) [32], Haemonchidae family (*Mecistocirrus digitatus*, NC_013848, *Haemonchus placei*, NC_029736) [19] and (*Haemonchus contortus*, NC_010383) [33], Dictyocaulidae family (*Dictyocaulus eckerti*, NC_019809; *Dictyocaulus viviparus*, NC_019810;) [34], whereas *Oesophagostomum quadrispinulatum* (GenBank accession number NC_014181) [23] was selected as an outgroup. The individual sequential alignment of amino acids derived from mt protein coding genes was performed using the MAFFT 7.122 software [35] and were chained into a single dataset. Furthermore, sequences that were aligned ambiguously were removed according to a previously described method [31]. Phylogenetic assessment was piloted using the neighbor joining (NJ), maximum likelihood (ML) and maximum parsimony (MP) methods using default parameters according to a formerly described method [36,37]. The bootstrap values for NJ and MP were 1000, whereas bootstrap 100 was selected for the ML analysis with a cutoff value of 95% for all methods. The number of differences model was used by NJ to infer the phylogenetic tree, and in the case of ML the uniform rates model was used. MP used the subtree-pruning-regrafting search method where the maximum trees to retain were 100. The FigTree v. 1.4 program (http://tree.bio.ed.ac.uk/software/figtree) was used to construct the phylograms.

## 3. Results and Discussion

### 3.1. ITS-2 Analysis

The obtained ITS-2 sequence had 99% identity to a previously published ITS-2 sequence of *O. trifurcata* (GenBank Accession no. AJ251124.1), suggesting that the worms collected are *Ostertagia trifurcata*.

### 3.2. Organization, Content and mt Genome Annotation

**Figure 1:**
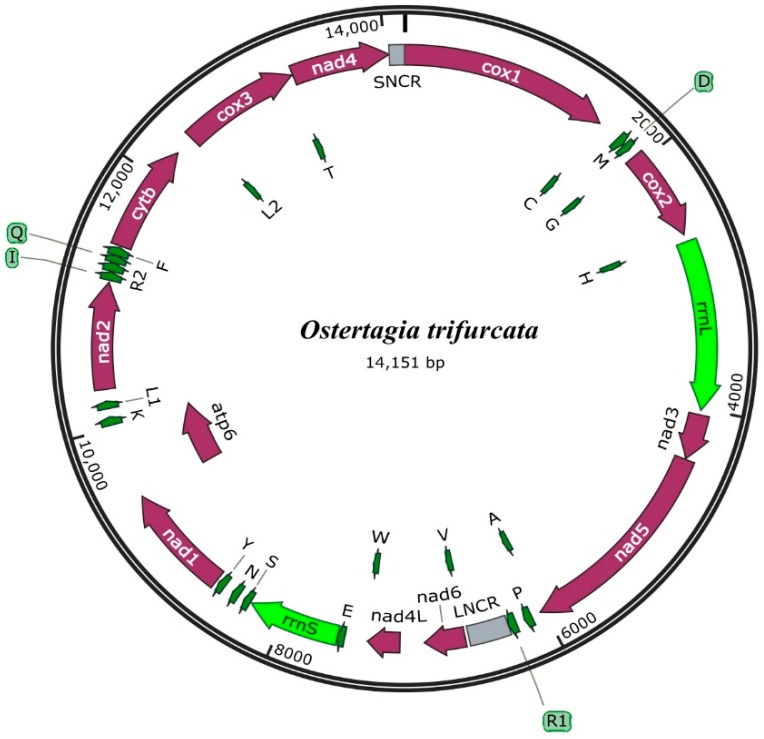
Mitochondrial genome arrangement of *Ostertagia trifurcata*. The scales are similar and all the genes are transcribed in the clockwise direction. The genes follow the standard nomenclature, except for the 22 tRNA genes, which are designated using one-letter amino acid codes with numerals differentiating each of the two leucine and arginine-specifying tRNAs (L1 and L2 for codon families UUR and CUN, respectively; R1 and R2 for codon families AGR and CGN, respectively). The tRNA genes and *atp*6 located on the inner circle indicate regions of gene overlap.

The complete mt genome of *O. trifurcata* (GenBank accession no. MK227249) was 14,151 bp in length (Figure 1). The mt genomes of Trichostrongyloidea published to date possess variations in size that range from 13,296 bp of *Dictyocallus eckerti* [34] to 15,221 bp of *Mecistocircus digitatus* [19]. The size of the *O. trifurcata* mt genome was found to be within the expected range, i.e., 14,151 bp. This mt genome includes 12 protein-coding genes (*nad*1-6, *cox*1-3, *cyt*b, *nad*4L and *atp*6), 2 rRNA genes, 22 tRNA genes and 2 non-coding regions (NC) (Table 2). The nucleotide composition of the coding strand of *O. trifurcata* was A = 4639 (32.78%), T = 6418 (45.35%), G = 2106 (14.88%) and C = 988 (6.98%). The gene contents and their organization were the same as those of *M. marshalli* [31], *D. viviparus* [34], *N. oiratianus* [22], *T. axei* [19] and *H. contortus* [33].

The mt genome of *O. trifurcata* encodes 12 proteins with 3181 amino acids. It accords three start codons (ATA, ATT, ATG) and two termination codons (TAA, TAG) (Table 2). Amongst the initiation codons, ATT was more frequently used, namely eight times by *cox*1, *cox*2, *nad*5, *nad*6, *nad*1, *atp*6, *nad*2 and *nad*4. ATA was utilized three times as the start codon by the *nad*3, *cyt*b and *cox*3 genes, whereas ATG was used once as the start codon for the *nad*4L gene. In the case of stop codons, TAA was most frequently used as the stop codon, namely ten times by the *cox*1, *cox*2, *cox*3, *nad*5, *nad*6, *nad*4L, *nad*1, *atp*6, *nad*2 and *cyt*b genes. The other stop codon was TAG, which was used by the *nad*3 and *nad*4 genes. These results are consistent with other studies of Trichostongyloidea nematodes (*T. circumcincta*, *T. axei* and *T. vitrinus*) [19], with some marked differences. In some previous mt genome studies of other nematodes of Trichostrongyloidea (*T. vitrines*, *T. axei* and *T. circumcincta*) [19], four start codons (ATA and TTG) were found, as well as incomplete stop codons (TA and A). However, in the present study, ATT and ATA were used as the initiation codons in the higher frequency by eleven protein coding genes and ATG was used once as a start codon. The present study also revealed the usage of complete termination codons as the stop codon. TAA was used altogether 10 times as the termination codon, and our data suggests the use of complete stop codons for all 12 genes coding for proteins. *O. trifurcata* is markedly different from other nematodes with regard to the basis of the start and stop codons, hence the provision of new molecular data provides insights into future studies of comparative mitochondrial genomics. Furthermore, the *O. trifurcata* mt genome possesses several overlaps between the CDS region and trnAs (Table 2). One nucleotide of *cox*1, *cox*2 and *nad*4 overlaps with *trn*C, *trn*H and *trn*T, respectively, whereas the *nad*1-*atp*6 and *trn*G-*cox*2 genes had overlaps of four and nine nucleotides, respectively. Moreover, there were longer overlaps in the mt genome sequence ranging from 20–50 nucleotides between *nad*4L–*trn*W, *atp*6–*trn*K, *trn*V–*nad*6, *cox*3–*trn*T, *nad*5–*trn*A, and *trn*L2 overlapping with the *cox*3 gene.

The *O. trifurcata* mt genome has 22 tRNA genes that range between 54 and 67 nucleotides in length. The *rrn*L gene of *O. trifurcata* is positioned between the *trn*H and *nad3* genes with a length of 1315 bp. The *rrn*S gene is situated between the two tRNA genes represented as *trn*E and *trn*S. The A+T content of both the rRNA genes is high, at 81.66% and 77.63%, respectively, for *rrn*L and *rrn*S (Table 3). The mt genome of *O. trifurcata* possesses two non-coding regions, represented as LNCR (large non-coding region) and SNCR (short non-coding region) (Table 2). The longer non-coding region (LNCR) is sited between the *trn*R1 gene and *trn*V with a length of 308 bp, whereas the shorter non-coding region (SNCR) is positioned between the *nad*4 and *cox*1 gene, with a length of 113 bp (Table 2). The A+T contents was found to be higher for both non-coding regions, at 80.19% and 76.10% for LNCR and SNCR, respectively. These non-coding regions might play a vital role in replication and transcription processes, however, the authentic processes are still unknown [38].

### 3.3. Phylogenetic Analysis

**Figure 2:**
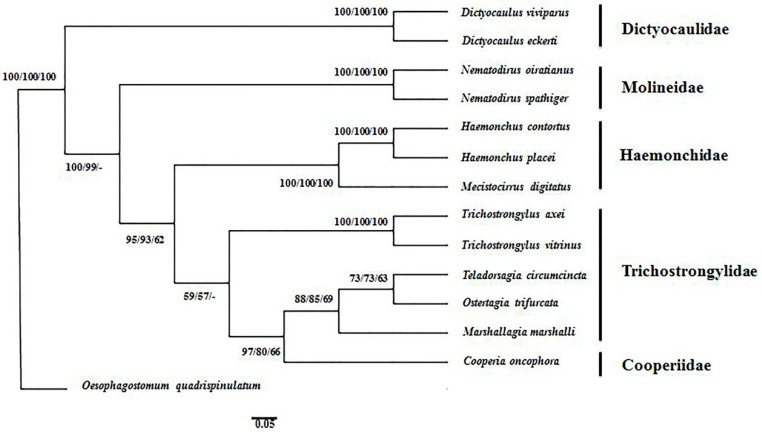
Phylogenetic tree inferred from concatenated amino acid sequences of 12 protein coding genes of key parasitic nematodes belonging to the Trichostrongyloidea family. Phylogenetic relationships of *Ostertagia trifurcata* and other members of Trichostrongyloidea were inferred using the neighbor joining (NJ), maximum likelihood (ML) and maximum parsimony (MP) methods. *Oesophagostomum quadrispinulatum* (GenBank accession number NC_014181) was chosen as the outgroup. The numbers along the branches indicate bootstrap values resulting from the analysis using NJ/ML/MP, where the values under 50 are given as “–”.

The sequences of amino acids of the 12 key representative nematodes belonging to the Trichostrongyloidea superfamily were concatenated to infer the phylogenetic tree (Figure 2) producing similar results using the maximum parsimony (MP), maximum likelihood (ML) and neighbor joining (NJ) methods. The results showed monophylies of Molineidae, Dictyocaulidae and Haemonchidae with significant statistical support, as shown in Figure 2. However, monophyly of the family Trichostrongylidae was rejected and these results were consistent with preceding studies [18,22,23]. Haemonchidae family species (*M. digitatus*, *H. contortus* and *H. placei*) were found to be more closely linked to the two species of the Trichstongylidae family compared to the other three species (*O. trifurcata*, *Teladorsagia circumcincta* and *Marshallagia marshalli*) belonging to the same family. The closer relationship within the Haemonchidae family was supported moderately (bootstrap values of NJ/ML/MP: 95/93/62, respectively) (Figure 2). Molineidae family species (*N. oiratianus* and *N. spathiger*) were found to be closer in evolutionary relationship to Dictyocaulidae family species (*D. viviparous* and *D. eckerti*) compared to *Trichostrongylus spp.*, (*O. trifurcata*, *T. circumcincta*) and Cooperidae (*C. oncophora*). The results in our study were consistent with earlier studies [22,31].

## 4. Implications and Significance

Gastrointestinal nematodes causing animal infections including ostertagiosis can sometimes be diagnosed on the basis of clinical presentation and symptoms such as chronic diarrhea, depressed appetite and high morbidity [1]. However, diagnosis only on the basis of clinical symptoms is usually unreliable as these symptoms can be present in animals with one or more gastrointestinal nematode members. The morphological identification of *O. trifurcata* is also not reliable enough at the larval stages. Fortunately, numerous DNA scientific methods have been developed as diagnostic tools for a number of nematodes [39,40,41,42]. The ITS-2 region has been used as a molecular marker for diagnosis and epidemiological investigation [43,44,45,46]. Therefore, the characterization of the mt genome of *O. trifurcata* now provides the basis for the development of innovative analytical and diagnostic tools as well as novel genetic markers.

The mt genome sequences, particularly sequences of protein coding genes have been used effectively for the systematic examination of the nematodes [9,17,27,47,48,49,50]. Consequently, we ascertained the mt genome of *O. trifurcata* in the current study, allowing a reassessment of systematic relationships using the datasets of Trichostrongyloidea nematodes. Regarding the members of Trichostrongyloidea (Trichostrongylidea, Cooperiidae, Haemonchidae, Molineidae and Dictyocaulidae), there have been disagreements about their systematic taxonomy. To date, the mt genomes of a number of species belonging to Trichostrongyloidea are not represented or are underrepresented. Therefore, expansion of the taxa sampling is very important to carrying out phylogenetic studies of Trichostrongyloidea species utilizing the mt genome datasets in the future.

## 5. Conclusions

The complete mt genome of *O. trifurcata* was determined in the present study. The molecular data presented in this study provides new mtDNA resources for the better consideration of phylogeny and mt genomics. It also provides useful and unique genetic markers for studying the diagnosis, molecular epidemiology, systematics and population genetics of *O. trifurcata* in small ruminants.

## Figures and Tables

**Table 1:** Sequences of primers used to amplify ITS-2 region and long fragments of mitochondrial DNA from *Ostertagia trifurcata.*

Primer	Sequence (5′ to 3′)	Region
NC5	GTAGGTGAACCTGCGGAAGGAT	ITS-2
NC2	TTAGTTTCTTTTCCTCCGCT	
37F	GGAGTAAAGTTGTATTTAAAC	*rrn*S-*cyt*b
36R	CCTCAAACTAAAACATAACC	
45F	ACTAGTTTGTTAAGTGTTATTCCT	*cyt*b-*ox*1
48R	ATAAACCTCAGGATGCCCAAAAAA	
CO1F	TTTTTTGGGCATCCTGAGGTTTAT	*cox*1-*rrn*L
40R	GAATTAAACTAATATCACGT	
39F	TAAATGGCAGTCTTAGCGTGA	*rrn*L-*rrn*S
4R	TCTACTTTACTACAACTTACTCC	

**Table 2:** Structure of the mitochondrial genome of *Ostertagia trifurcata* and nucleotide positions of the starting and termination sites as well as the length of each gene and the number of encoded amino acids, starting and terminator codons of protein coding genes and anticodons for tRNAs starting from *trn*C.

Gene/codons	Position and sequence length of nt	Amino acids	Start/stop codons	Anticodons
*cox*1	1–1578 (1578)	525	ATT/TAA	
*trn* C	1578–1634 (57)			GCA
*trn* M	1753–1813 (61)			CAT
*trn* D	1823–1876 (54)			GTC
*trn* G	1893–1948 (56)			TCC
*cox*2	1937–2644 (708)	235	ATT/TAA	
*trn* H	2644–2699 (56)			GTG
*rrn*L	2700–4014 (1315)			
*nad*3	4048–4398 (351)	116	ATA/TAG	
*nad*5	4405–6030 (1626)	541	ATT/TAA	
*trn* A	5986–6044 (59)			TGC
*trn* P	6102–6162 (61)			TGG
*trn* R_1_ (AGR)	6227–6288 (62)			TCT
LNCR	6289–6596 (308)			
*trn* V	6597–6653 (57)			TAC
*nad*6	6623–6928 (306)	101	ATT/TAA	
*nad*4L	7110–7352 (243)	80	ATG/TAA	
*trn* W	7332–7389 (58)			TCA
*trn* E	7523–7577 (55)			TTC
*rrn*S	7578–8270 (693)			
*trn* S	8277–8330 (54)			TGA
*trn* N	8376–8431 (56)			GTT
*trn* Y	8502–8556 (55)			GTA
*nad*1	8578–9432 (855)	284	ATT/TAA	
*atp*6	9428–10045 (618)	205	ATT/TAA	
*trn* K	10025–10088(64)			TTT
*trn* L_1_ (UUR)	10153–10210 (58)			TAA
*nad*2	10302–11126 (825)	274	ATT/TAA	
*trn* I	11141–11205 (65)			GAT
*trn* R_2_ (CGN)	11211–11277 (67)			ACG
*trn* Q	11280–11336 (57)			TTG
*trn* F	11337–11403 (67)			GAA
*cyt*b	11415–12251 (837)	278	ATA/TAA	
*trn* L_2_ (CUN)	12435–12490 (56)			TAG
*cox*3	12401–13294 (894)	297	ATA/TAA	
*trn* T	13244–13301 (58)			TGT
*nad*4	13301–14038 (738)	245	ATT/TAG	
SNCR	14039–14151 (113)			

*LNCR—large non-coding region, SNCR—short non-coding region.

**Table 3:** Composition of nucleotides and skew values of *Ostertagia trifurcata* mitochondrial protein-coding genes.

Gene	A	G	C	T	A+T (%)	AT skew	GC skew
*cox*1	25.98	20.08	10.89	43.02	69.00	−0.24	−0.29
*cox*2	31.35	17.37	8.61	42.65	74.00	−0.15	−0.33
*nad*3	33.33	13.96	3.70	49.00	82.33	−0.19	−0.58
*nad*5	31.54	13.71	6.39	48.33	79.87	−0.21	−0.36
*nad*6	27.12	14.05	4.90	53.92	81.04	−0.33	−0.48
*nad*4L	32.09	16.87	2.46	48.55	80.64	−0.20	−0.74
*nad*1	25.84	17.66	7.95	48.53	74.37	−0.30	−0.37
*atp*6	28.96	17.31	6.14	47.57	76.53	−0.24	−0.47
*nad*2	30.42	11.63	5.21	52.72	83.14	−0.26	−0.38
*cyt*b	27.83	18.87	9.67	43.84	71.67	−0.22	−0.32
*cox*3	28.63	16.55	7.60	47.20	75.83	−0.24	−0.37
*nad*4	29.13	13.41	7.04	50.40	79.53	−0.26	−0.31
*rrn*L	37.79	12.24	6.08	43.87	81.66	−0.07	−0.33
*rrn*S	36.65	14.71	7.64	40.98	77.63	−0.05	−0.31
LNCR	37.66	16.88	2.92	42.53	80.19	−0.06	−0.70
SNCR	32.74	10.61	13.27	43.36	76.10	−0.13	−0.11
Overall	32.78	14.88	6.98	45.35	78.13	−0.16	−0.36
